# DeepChestGNN: A Comprehensive Framework for Enhanced Lung Disease Identification through Advanced Graphical Deep Features

**DOI:** 10.3390/s24092830

**Published:** 2024-04-29

**Authors:** Shakil Rana, Md Jabed Hosen, Tasnim Jahan Tonni, Md. Awlad Hossen Rony, Kaniz Fatema, Md. Zahid Hasan, Md. Tanvir Rahman, Risala Tasin Khan, Tony Jan, Md Whaiduzzaman

**Affiliations:** 1 Health Informatics Research Laboratory (HIRL), Department of Computer Science and Engineering, Daffodil International University, Dhaka 1207, Bangladesh; shakil15-3816@diu.edu.bd (S.R.); hosen15-3834@diu.edu.bd (M.J.H.); tasnim15-3789@diu.edu.bd (T.J.T.); awlad15-12208@diu.edu.bd (M.A.H.R.); kaniz15-12344@diu.edu.bd (K.F.); zahid.cse@diu.edu.bd (M.Z.H.); 2 School of Health and Rehabilitation Sciences, The University of Queensland, St. Lucia, QLD 4072, Australia; 3 Department of Information and Communication Technology, Mawlana Bhashani Science and Technology University, Tangail 1902, Bangladesh; 4 Institute of Information Technology, Jahangirnagar University, Dhaka 1342, Bangladesh; risala@juniv.edu; 5 Centre for Artificial Intelligence Research and Optimisation (AIRO), Torrens University, Ultimo, NSW 2007, Australia; tony.jan@torrens.edu.au; 6 School of Information Systems, Queensland University of Technology, Brisbane, QLD 4000, Australia

**Keywords:** chest X-ray images, deep convolutional neural network, elastic deformation, feature extraction, graph neural network, image pre-processing, lung disease

## Abstract

Lung diseases are the third-leading cause of mortality in the world. Due to compromised lung function, respiratory difficulties, and physiological complications, lung disease brought on by toxic substances, pollution, infections, or smoking results in millions of deaths every year. Chest X-ray images pose a challenge for classification due to their visual similarity, leading to confusion among radiologists. To imitate those issues, we created an automated system with a large data hub that contains 17 datasets of chest X-ray images for a total of 71,096, and we aim to classify ten different disease classes. For combining various resources, our large datasets contain noise and annotations, class imbalances, data redundancy, etc. We conducted several image pre-processing techniques to eliminate noise and artifacts from images, such as resizing, de-annotation, CLAHE, and filtering. The elastic deformation augmentation technique also generates a balanced dataset. Then, we developed DeepChestGNN, a novel medical image classification model utilizing a deep convolutional neural network (DCNN) to extract 100 significant deep features indicative of various lung diseases. This model, incorporating Batch Normalization, MaxPooling, and Dropout layers, achieved a remarkable 99.74% accuracy in extensive trials. By combining graph neural networks (GNNs) with feedforward layers, the architecture is very flexible when it comes to working with graph data for accurate lung disease classification. This study highlights the significant impact of combining advanced research with clinical application potential in diagnosing lung diseases, providing an optimal framework for precise and efficient disease identification and classification.

## 1. Introduction

Lung diseases comprise a variety of conditions that impact the functionality of the lungs and respiratory system, which can lead to impaired lung function and breathing. A wide range of bacterial, viral, or fungal infections can cause them [1]. Environmental factors have been linked to various lung diseases, including asthma, mesothelioma, and lung cancer, which affect respiratory health, including chronic obstructive pulmonary disease (COPD) and infections such as influenza, pneumonia, and tuberculosis [2]. Lung disease causes persistent shortness of breath, mucus production, coughing, blood coughing, close contact, sneezing, coughing, respiratory droplets, and unexplained chest pain lasting over a month [3]. These symptoms are early indicators of respiratory problems and underscore the necessity of medical evaluation and care. According to the World Health Organization (WHO), 3.23 million people died from COPD in 2019. As a result, it is the seventh leading cause of death worldwide. In addition, over 70% of COPD cases in high-income countries are caused by smoking, while 30–40% in low-income countries are caused by household air pollution [4].

Nowadays, COVID-19, a concerning lung disease, leads to fluid-filled air sacs, respiratory infections, and cold-like symptoms [5]. Pneumonia fills the alveoli with pus or fluid, pneumothorax causes lung collapse with air escape, and effusion is excess fluid outside the lung [6]. On the other hand, pulmonary fibrosis results from lung damage, tuberculosis creates lung cavities, and lung opacity indicates damaged areas. Lung masses and nodules are irregular growths like tumors [5]. Screening for lung abnormalities is necessary to detect this common lung disease. Imaging techniques such as chest X-rays, computed tomography (CT), magnetic resonance imaging (MRI), positron emission tomography (PET) scans, and echocardiograms are essential for diagnosing lung diseases [7]. Chest X-rays are cost-effective, user-friendly, and faster than CT scans and other diagnostic techniques, providing extensive patient information [8]. Medical professionals widely use X-rays to diagnose various conditions, including fractures, cancer, pneumonia, and dental issues. They provide insights into lung structure and function, aiding early detection and effective treatment [9]. Also, radiologists can make subjective assessment errors or cannot detect disease easily, causing unclear and abnormal interference in chest radiographic images. As a result, the patient suffers from lengthy diagnostic procedures and increased radiation exposure. Addressing these challenges and enhancing clinical diagnosis, we employed an automated system for chest radiographic images, which can efficiently diagnose respiratory issues. Numerous recently developed automated systems were examined in our literature review [5,9,10,11,12,13,14,15,16,17,18,19,20,21]. Challenges were encountered by those systems in data handling during image processing and optimal feature extraction, complicated quantification, and high runtime complexity issues in classifying chest X-ray images. Furthermore, the existing automated system, with such an extensive, large data hub with a significant ten-type class classification on chest X-rays, was not utilized. Additionally, their computational time is high due to handling numerous features, leading to time complexity issues.

In this study, we established one of the most extensive publicly available datasets [22,23,24,25,26,27,28,29,30,31,32,33,34,35,36,37,38], containing 17 datasets of chest X-ray images sourced from GitHub, Kaggle, Mendeley Data, and the National Institutes of Health (NIH) Clinical Centre. The ten disease categories in the large data hub are normal, effusion, pulmonary fibrosis, lung opacity, mass, nodule, COVID-19, pneumonia, pneumothorax, and tuberculosis. A deep convolutional neural network (DCNN) was proposed to analyze images and extract optimal deep features, followed by the proposal of a graph neural network (GNN) model named DeepChestGNN employing the deep features and optimizing the model, exhibiting improved accuracy without compromising efficiency in categorization, making it more time-effective than the base model. Various image pre-processing techniques were employed, including resizing, denoising, CLAHE, de-annotation, and filtering. Integration of advanced image enhancement and robust machine learning techniques led to superior performance in lung issue diagnosis. Additionally, elastic deformation was utilized for dataset augmentation, leveraging a substantial dataset of 70,000 images to enhance learning. The feature extractor model, DCNN, effectively extracted 100 crucial details using specialized layers and techniques. The proposed DeepChestGNN also went through a lot of testing using different parameters in the ablation study, which made its structure and learning process better. Our model has a well-organized structure with batch normalization, MaxPooling, and Dropout for regularization, finding a better balance between being expressive and not overfitting. This made it possible to classify the large hub of chest X-ray images in a lot less time with low time complexity. Comprehensive quality control measures ensured accurate disease detection compared to other methods. Achieving outstanding performance involved extensive data preparation, meticulous model design, and fine-tuning, resulting in clear, standardized images with enhanced details facilitating accurate diagnosis.

The primary contributions of this research can be summarized as follows:

- In this study, we have collected around 17 raw chest X-ray datasets from different sources and combined them into a single comprehensive dataset. Images vary in quality since they are compiled from several different sources. Consequently, the final dataset comprises ten different lung disease images.
- Effective image pre-processing techniques improve lung disease classification accuracy by reducing noise and artifacts. In this regard, we resized all images to the same pixel. The de-annotation method improves images by removing annotations and extraneous text, and the enhancement method enhances image properties. We used elastic deformation methods to add random distortions to the data to balance the distribution of underrepresented class images.
- We have created a novel DCNN architecture that extracts the 100 prominent deep features from X-ray images through a strategic architecture incorporating batch normalization, MaxPooling, and dropout layers. Its deep structure captures intricate patterns, crucial for advanced medical image analysis, highlighting its efficacy in feature representation.
- A proposed DeepChestGNN is built on an efficient bi-layered architecture incorporating graph convolutional and feedforward layers. This architecture, informed by rigorous ablation examinations and fine-tuned through hyperparameter research, demonstrates GNN’s flexibility to graph data. These layers were added to fulfill the difficulty of identifying and classifying lung diseases.

## 2. Literature Review

Several studies have been conducted in recent years to diagnose lung diseases based on X-ray images using various deep learning and machine learning techniques. So, we reviewed several papers where researchers explored different classification methods using X-ray images. However, all the studies are demonstrated in this section.

Sanida et al. [10] introduced a new deep learning (DL) framework for lung disease diagnosis using chest X-ray images (21,165 chest X-ray images). They employed the modified VGG19 model for multi-class classification (fibrosis, opacity, tuberculosis, normal, viral pneumonia, and COVID-19 pneumonia) and achieved an accuracy of 98.88%. However, their main limitations were the lack of noise reduction, overlay text removal from images, and a limited number of datasets. Meanwhile, Abubakar et al. [11] discussed using medical imaging techniques and machine learning methods for early COVID-19 diagnosis using three CT image datasets (328 images of common pneumonia, 1972 images of COVID-19, and 1608 images of healthy images). They extracted features using eight deep learning models. The combination of the histogram of oriented gradients (HOG) and deep learning features, specifically VGG-16, achieved the highest overall accuracy of 99.4% with the SVM classifier for multi-class classification. The absence of image pre-processing, including noise reduction and text overlay removal, and the limited number of images were noted limitations.

In another work, Kufel et al. [12] utilized a large dataset named NIH ChestX-ray 14 along with 112,120 images. Their model employed EfficientNet for feature extraction, using transfer learning techniques. This multi-class classification method obtained an accuracy of 84.28% in classifying fifteen chest pathology classes. The absence of image pre-processing in this work is a significant limitation that results in reduced multi-class classification accuracy. To address multi-class classification problems, Li et al. [13] utilized the same ChestX-Ray 14 and CheXpert medical image classification datasets. They used a Multi-Level Residual Feature Fusion Network (MLRFNet) classifier and a Res2Net50 feature extractor to achieve such great results. The task involves categorizing images into seven different classes. The ChestX-Ray 14 dataset achieved an accuracy of 85.30%, and the CheXpert dataset achieved 90.40% accuracy. The primary limitations include the lack of image pre-processing, the absence of information on optimal features, and low accuracy in multi-class classification. Meanwhile, Farhan and his colleagues [14] looked into how to diagnose COVID-19 pneumonia using the COVID-19 Radiography Database (C19RD), which contains 2905 images, and the Chest X-ray Images for Pneumonia (CXIP) dataset, consisting of 5856 images. In particular, they used the ResNet50 feature extractor and the Hybrid Deep Learning Algorithm (HDLA-DNN) to differentiate between disease classes (e.g., non-COVID-19 pneumonia or COVID-19 pneumonia) and healthy classes. The outcomes are noteworthy, showcasing an impressive accuracy of 98.35% and 98.99% for the C19RD and CXIP datasets, respectively. Nevertheless, the primary limitation of this paper was the lack of multi-class classifications, as it only performed binary class classification. Nahiduzzaman and his co-workers [15] conducted research using around 29,871 images of the ChestX-Ray14 dataset. They applied the extreme learning machine algorithm (ELM) as a feature extractor within the CNN-ELM algorithm. The study aimed to classify 17 lung diseases, including COVID-19 and tuberculosis, with an overall accuracy of 90.92%. It achieved an accuracy of 99.37% for COVID-19 and 99.98% for tuberculosis. The study’s limitations include a lack of proper augmentation technique, absence of information on optimal features, and low accuracy in multi-class classification.

Moreover, another study by Jin et al. [16] worked with a sizable ChestX-ray 14 dataset that included 112,120 images. They used DenseNet121 to extract features and built the Cross-Modal Deep Metric Learning Generalized Zero-Shot Learning (CM-DML-GZSL) classifier, which combines graph convolutional networks (GCNs) and 3D-CNN to classify images by disease. Despite a complex methodology, the study attained a classification accuracy of 80%, with a significant limitation being low accuracy in multi-class classification and limited class classifications. Even though Tang and his co-workers [17] used two substantial datasets, a chest X-ray (CXR) dataset comprising 6939 images and a CT dataset containing a notable 85,725 images, a Node-Self Convolution Graph Convolutional Network (NSCGCN) and a DenseNet201 feature extractor were employed to classify diseases into two classes: infection and normal. Their method obtained excellent accuracy rates of 97.09% for the CXR dataset and 99.22% for the CT dataset. Nevertheless, the limitations were the lack of comprehensive image pre-processing, which might help improve feature extraction and model performance. In a study by Shamrat et al. [5], 85,105 chest X-ray images from diverse sources were utilized, covering ten classification classes. They employed eight pre-trained CNN models: AlexNet, GoogLeNet, InceptionV3, MobileNetV2, VGG16, ResNet50, DenseNet121, and EfficientNetB7. The model achieved accuracies of 92.95% and 98.89% for VGG16 and LungNet22, respectively. Nevertheless, the study emphasizes the limitation of the absence of optimal feature extraction.

In another study, Guail et al. [9] focused on leveraging a chest X-ray dataset comprising 5856 images from Kaggle. First, they used CNN to extract features. Then, they employed a Principal Neighbor Aggregation-Based Graph Convolutional Network (PNA-GCN) for binary classification tasks, such as distinguishing between people who had pneumonia and those who were healthy. The outcomes of this study demonstrated a commendable accuracy rate of 97.79%. However, there are several notable limitations, such as a limited number of images, a need for proper augmentation techniques, and limited class classifications. Furthermore, Ragab et al. [18] used 6310 chest X-ray images from Kaggle. They initially extracted CNN features and then employed the Capsule Neural Network (CapsNet) model for multi-class classification, including pneumonia, average, and COVID-19. They achieved an accuracy of 86.6% for the standard class, 94% for the pneumonia class, and 89% for the COVID-19 class. The primary limitations of this study encompass limited images, no proper augmentation, a lack of optimal feature information, and no multi-class classification. Liang et al. [19] concentrated on binary classification using a dataset of 399 COVID-19 and 400 normal images. The study employed a 3D-CNN for feature extraction and utilized GCN as the classifier, achieving an impressive accuracy of 98.5%. Nevertheless, this study has a limited number of images, no proper augmentation technique, no optimal feature technique, and no multi-class classification. Moreover, Javaheri et al. [20] leveraged an expansive dataset comprising 16,750 slices from various CT scan images to develop CovidCTNet, an open-source framework to enhance the accuracy of CT imaging detection. The model achieved notable results with 93.33% accuracy in binary classification (distinguishing between COVID-19 and non-COVID-19 cases) and 86.66% accuracy in multi-class classification (including COVID-19, CAP, and control lungs). However, the study’s primary limitations lie in the absence of image pre-processing and the relatively lower accuracy observed in multi-class classification tasks. Alshazly et al. [21] introduced novel deep learning methodologies for automated COVID-19 detection, leveraging two distinct CT image collections: the SARS-CoV-2 CT Scan dataset (comprising 2482 CT scans) and the COVID-19-CT dataset (consisting of 746 CT images). Among the models evaluated, ResNet101 demonstrated superior performance across various evaluation metrics on the SARS-CoV-2 CT dataset, achieving an impressive average accuracy and F1-score of 99.4%. However, a notable drawback in the study is the lack of image pre-processing, such as noise reduction and text overlay removal, compounded by the relatively small dataset size.

After reviewing all the literature, it can be noted that there is a scarcity of applying proper image pre-processing, augmentation techniques, and optimal feature extraction to the large data hub. Moreover, their model obtained a low accuracy in multi-class classification and a limited number of class categories. However, Table 11 shows all the limitations. Since their work has several significant limitations, we have addressed them by introducing various advanced methods to improve accuracy in terms of image classification significantly. For a better understanding, all the processes are described in detail below.

## 3. Materials and Methods

**Figure 1:**
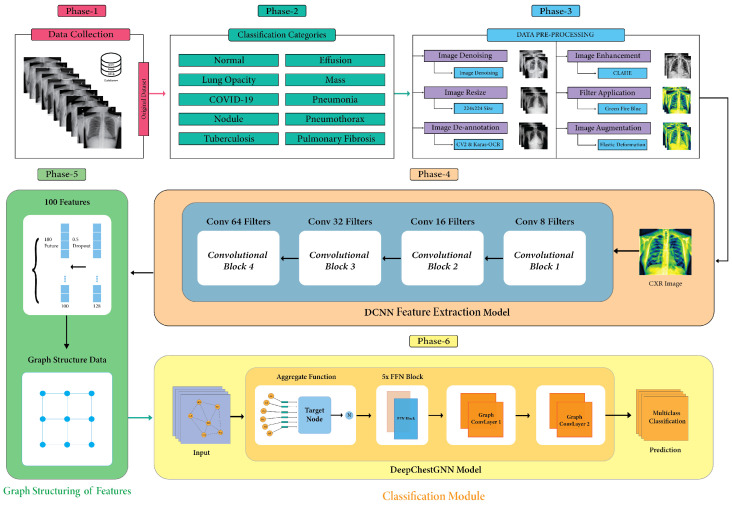
Main workflow diagram.

This study aims to introduce an automated system for categorizing chest X-rays according to different diseases. In phase-1, initially, we collected data from various resources; in phase-2, we addressed ten different diseases on chest X-rays; in phase-3, we performed different image pre-processing techniques to resize images with the same pixels, remove distracting text and noise, and improve contrast. We used elastic deformation to improve well-balanced datasets; in phase-4, we suggested a DCNN model that pulls out the most important deep features; in phase-5, we used graph structure to make a good dataset for the model; and in phase-6, we suggested a DeepChestGNN model to diagnose diseases. Figure 1 visually represents the main workflow diagram according to phase.

### 3.1. Dataset Description

**Figure 2:**
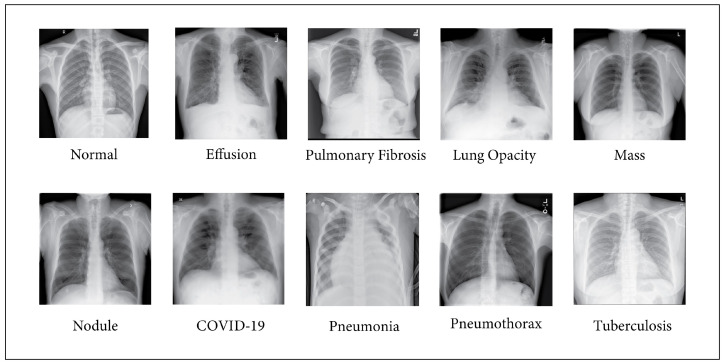
Images of ten different classes.

In this paper, we have collected numerous chest X-ray images from multiple sources and created one of the most extensive publicly available datasets. After collecting them from various database sources, we merged them to create a large data hub for classifying diseases. As part of a collective dataset, our data hub comprises 17 chest X-ray images, totaling 71,096. Table 1 presents the compiled information corresponding to the data sources collected for each class. The dataset consists of ten chest X-ray images, each characterized by a specific number of images. Notably, the “Normal” class contains 13,953 images and is collected from labeled datasets [22,23,26,27,29,36]. Additionally, the “Tuberculosis” class includes 5242 images and is sourced from datasets [22,30,32,36], while “Lung Opacity” consists of 7236 images drawn from datasets [24,29]. A total of 11,566 images from datasets [24,26,27,29,31] make up the “COVID-19” class, and 11,683 images from datasets [23,24,26,32] make up “Pneumonia”. The “Pneumothorax” class comprises 6148 images gathered from datasets [25,33,37], and “Nodules” includes 4131 images from datasets [25,28,38]. “Fibrosis” contains 2821 images and is collected from datasets [25,28,32], while “Effusion” encompasses 5557 images sourced from datasets [25,28,30,35]. Last but not least, 2903 images from datasets [25,28] represent the “Mass” class. The combined dataset consists of ten classes of images, as illustrated in Figure 2.

### 3.2. Image Preprocessing

Image preprocessing of medical images is crucial before adding images to a neural network, impacting accuracy significantly [39,40]. The proposed image pre-processing technique stood out from existing methods. Sanida et al. [10], Abubakar et al. [11], and Ragab et al. [18] mainly focused on resizing images. Guail et al. [9] used augmentation, and Nahiduzzaman et al. [15] emphasized resizing and normalization, often insufficient. Farhan et al. [14] applied the Wiener filter, which was effective for noise reduction but struggled with detail preservation and adaptability. Motivated by their methods, we used different approaches that include several steps: denoising, resizing, de-annotation, enhancement, and filter application. This process removes artifacts, minimizes noise, and emphasizes significant objects.

#### 3.2.1. Image Resizing

At first, all the images were resized to 224 × 224 pixels since the included images have different pixel dimensions. This standardization was necessary due to variations in pixel dimensions across the included images, as we integrated extensive datasets from diverse sources [41].

#### 3.2.2. Image Denoising

Due to the limitations of imaging sensors and the circumstances of the surrounding environment, noise is a problem that is impossible to avoid in digital images. To address this problem, we developed a total variation (TV) denoising algorithm called the denoise_tv_chambolle technique, which is based on the idea that images containing erroneous information, which may be incorrect, have large total variations [42]. *a* denotes the predicted image, a∈Ω∈R, and the regularization term $\int_{\Omega }\left|\Delta a\left(x\right)\right|\;dx$ denotes the prior constraint of image *a*, which is used for image denoising. The whole equation is defined as:

(1) $$TV\left(a,\Omega \right)=\int_{\Omega }\left|\Delta a\left(x\right)\right|\;dx$$

**Figure 3:**
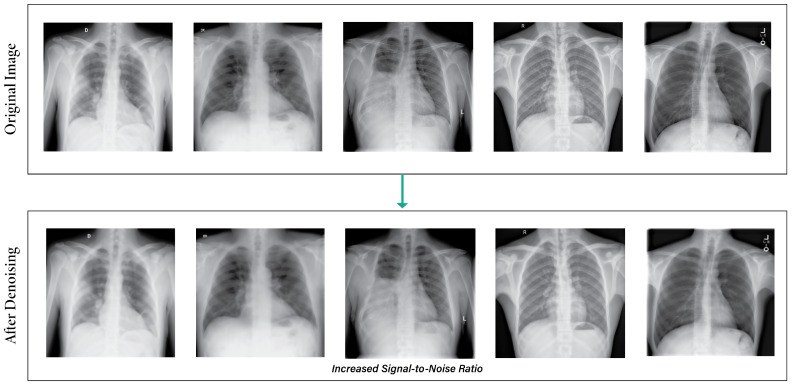
Output images after applying the denoising technique.

Figure 3 illustrates the output images after applying the denoising images. The power of total variation regularization is harnessed in this method to reduce noise and improve image quality without altering critical features like edges and structures [43].

#### 3.2.3. De-Annotation

**Figure 4:**
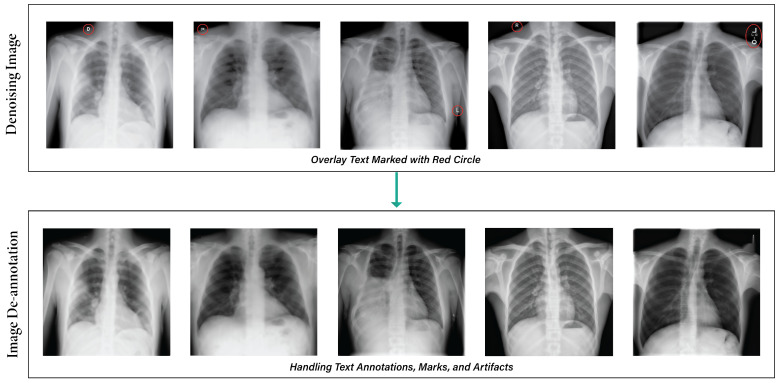
Output after applying the de-annotation method.

Image annotations can be challenging in certain datasets, especially if they are unnecessary or hinder research. Image text eraser removes text from images and replaces it with a natural backdrop, leaving non-text parts alone [5]. Figure 4 depicts the results after applying the de-annotation method.

Our medical image de-annotation preprocessing was performed using approaches such as CV2 and Keras-OCR. The Keras-OCR algorithm is a pre-trained OCR model that automatically removes text from images without needing a specific model [44]. It utilizes a mask on bounding boxes to indicate the particular region for inpainting, thereby maintaining visual continuity in medical imaging. The algorithm performs well when text boxes are close to other objects. Nevertheless, it may perform poorly when a text box is close to other objects [45].

#### 3.2.4. Image Enhancement

The contrast of our images was enhanced by the utilization of contrast-limited adaptive Histogram Equalization (CLAHE), effectively mitigating the issue of noise amplification, a prevalent limitation seen in traditional histogram equalization methods [5]. The CLAHE technique was designed to increase the quality of low-contrast medical images [46]. The amplification in CLAHE is restricted by performing a clipping operation on the histogram at a user-defined value known as the clip limit [47]. The adjustment of the clipping level determines the extent to which noise within the histogram is subjected to smoothing, influencing the degree of contrast enhancement [48]. A color version of CLAHE was employed in our study. We maintained the tile grid size at (8 × 8) and the clipping limit at 3.0 [49]. The steps for applying CLAHE are as follows:

- Initially, the RGB image was transformed into an LAB image.
- Subsequently, the CLAHE approach was employed to enhance the L channel.
- Next, the enhanced L channel should be paired with the A and B channels to obtain an enhanced LAB image.
- Eventually, the improved LAB image was converted to revert to its original form as the enhanced RGB image.

The CLAHE method partitions an image of size P×P into blocks of size p×p. Each block’s histogram is computed, and a threshold, $TL=L_{\mathrm{norm}}\times \frac{X\times Y}{G}$, is set, with TL=0.002. Pixels of grayscale level *K* exceeding TL are clipped, with an average of $\frac{L_{\mathrm{prune}}}{G}$ pixels distributed across levels. The redistribution is $L_{\mathrm{dist}}=\frac{G}{L_{\mathrm{residual}}}$ post-distribution, and the histogram is equalized. Artificial edges are mitigated via bi-linear interpolation [50].

(2) $$I_{\mathrm{adj}}\left(q,r\right)=\frac{\left(Z-1\right)}{PQ}\sum_{k}f_{k}$$

**Figure 5:**
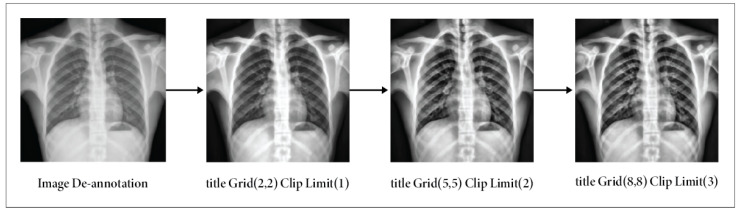
Outputs of applying CLAHE on de-annotation images: results using Clip Limit (3) & TileGridSize (8, 8).

$I_{\mathrm{adj}}\left(q,r\right)$ refers to the adjusted pixel intensity. From normalization, it includes the maximum intensity (Z−1) throughout the image’s domain size PQ and the frequencies of various intensity levels *k*. Figure 5 illustrates how the contrast enhancement maintains the image elements’ suitability.

#### 3.2.5. Filter Application

**Figure 6:**
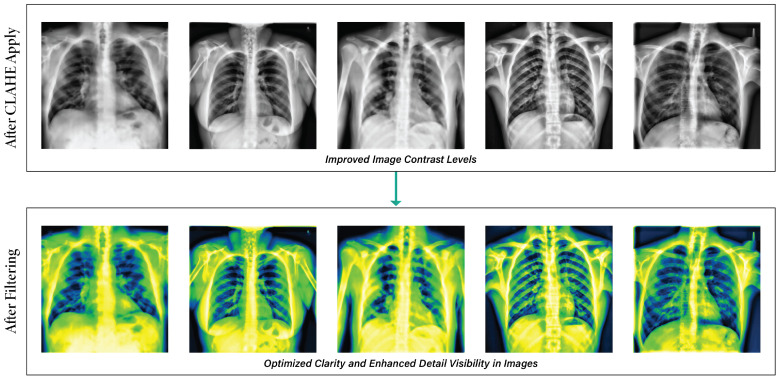
Output of using the Green Fire Blue filter on the CLAHE images.

Filtering techniques are essential in enhancing X-ray image features, optimizing them for training, validation, and testing objectives. Several image enhancement techniques utilize median filters to enhance the information in images [51], and in this instance, we applied the ‘Green Fire Blue’ filter, which offered a distinct visual perspective by emphasizing specific image attributes that were previously marginalized or muted [5]. Figure 6 illustrates the way the filtering method is used on the clear and enhanced images.

**Figure 7:**
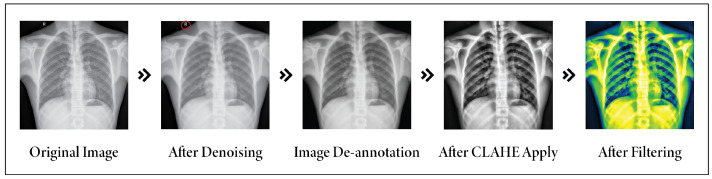
Total image pre-processing methods.

After addressing all the noise, challenges, and unwanted text in images, preprocessing techniques play a crucial role in substantially enhancing the quality and interpretability of medical images. Figure 7 illustrates the result of all the preprocessing steps for an image, including de-noising, annotation removal, CLAHE, and fitting.

### 3.3. Elastic Deformation Augmentation

Traditional data augmentation techniques, such as rotation, flipping, and rescaling, have proven ineffective in capturing biological variability in medical image data. This limitation arises from the fact that the shapes of biological tissues experience elastic deformations when subjected to compression from adjacent organs [52]. This method uses an external force to increase the elasticity of materials, thereby boosting their performance and longevity. It may replicate the tissue’s appearance and represent changes in form [53]. Two matrices are used in the elasticity deformation technique; $E_{x}$ and $E_{y}$ are designed to record the distances between each pixel along the *x*-axis and *y*-axis, respectively.

First, each point is either moved randomly for a distance of *d* or it remains unmoved.

(3) $${\mathrm{Ex}}_{ij},{\mathrm{Ey}}_{ij}\in \left\{-d,0,d\right\}$$

**Figure 8:**
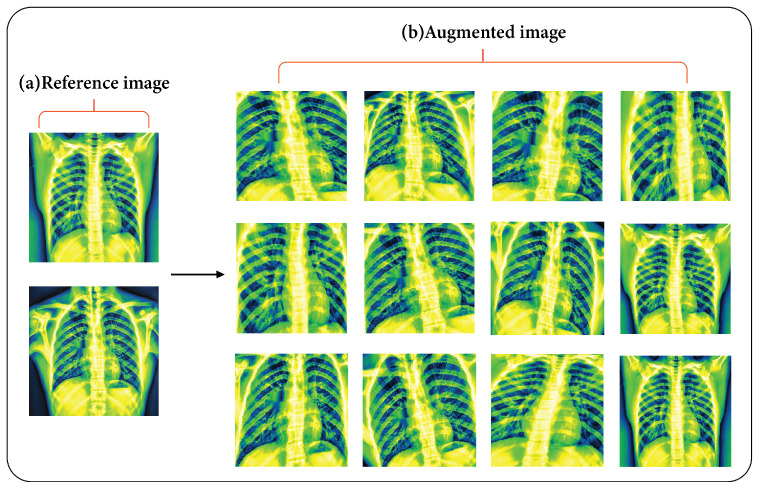
(**a**) Reference image and (**b**) twelve augmented images after applying the elastic deformation method.

After that, each of the two matrices, two one-dimensional Gaussian kernels of size *k* (*k* should be an odd number), and standard deviation σ are added [48]. Each row of the matrices $E_{x}$ and $E_{y}$ is filtered with the initial Gaussian kernel $G_{x}$:

(4) $$A_{x}=\alpha *e^{-{\left(x-\left(k-1\right)/2\right)}^{2}/\left(2*\sigma^{2}\right)}$$

where x=0,…,k−1 and α is the scale factor chosen so that $\Sigma_{x}$$G_{x}=1$. Then every column of $E_{x}$ and $E_{y}$ is filtered with the second Gaussian kernel $G_{y}$:

(5) $$A_{y}=\alpha *e^{-{\left(x-\left(k-1\right)/2\right)}^{2}/\left(2*\sigma^{2}\right)}$$

where y=0,…,k−1 and α is the scale factor chosen so that $\Sigma_{x}$$G_{x}=1$. Finally, each pixel of the original image is moved according to the distances in $E_{x}$ and $E_{y}$. Data augmentation was accomplished through the use of various combinations of *k* and σ. Figure 8 shows the outputs of the augmentation methods.

Regardless of the application’s completion of these various processing techniques, every class now contains 7000 images. Table 2 shows the total number of 70,000 images in the dataset.

### 3.4. Proposed Model

#### 3.4.1. Feature Extraction Model

Feature extraction was crucial for identifying key attributes within medical image data, significantly enhancing the performance of the deep learning model, specifically designed as a CNN architecture. The CNN architecture, including convolutions, batch normalization, and maximum pooling, was created to handle complexity and avoid overfitting, enhancing model generalization [54]. Our DCNN architecture introduces unique features that distinguish it from existing models. We have improved our architecture compared to other CNNs [9], modified VGG19 [10], EfficientNet [12], CNN-ELM [15], DenseNet121 [16], and DenseNet201 [17]. This is because it uses new connection patterns and optimization strategies, which makes it better at many tasks and datasets. In this study, we employed a meticulously designed DCNN that is specifically engineered to extract features from X-ray images with high efficiency. The architecture of the DCNN comprises four Convolution Blocks (Conv_Block), each incorporating convolutional, batch normalization, and rectified linear unit (ReLU) activation layers [55]. The feature extraction process within the DCNN follows a systematic flow:

(6) $$Z_{i}\left(x\right)=A_{i-1}*W+b_{i}$$

(7) $$A_{i}=f_{\mathrm{pool}}\left(Z_{i}\right)$$

Within each feature extraction module, *Z* denotes the linear output of the convolutional layer, *A* represents the activation output of the preceding layer, *W* encompasses the weights associated with the convolutional kernel, *b* signifies the bias of the convolutional layer, and $f_{\mathrm{pool}}$ denotes the pooling function. Following the convolutional computation, the resulting features undergo ReLU activation before proceeding to the subsequent layer of connected units. This process iterates twice before the features are finally passed through the pooling layer for further processing.

**Figure 9:**
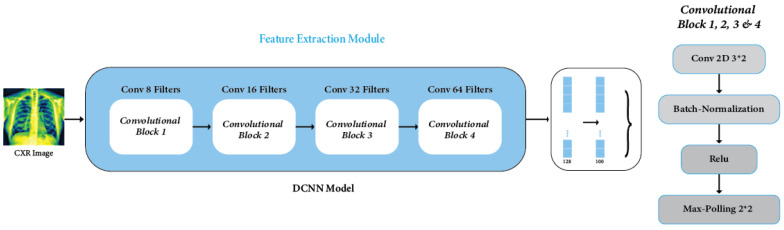
DCNN feature extraction module.

Additionally, in the DCNN architecture, a particular layer (outer ReLU layer) is strategically chosen for feature extraction [56]. The output from this layer is subsequently fed into the GNN classifier for accurate classification. Through the synergistic integration of convolutional and pooling layers, the DCNN progressively abstracts and comprehends high-level features inherent in the data [57]. This process enables a hierarchical understanding and extraction of intricate patterns essential for accurate classification tasks. The model’s generalization capabilities were significantly boosted through optimization using the Adam method across 100 epochs, a learning rate of 0.001, and a dropout rate of 0.50, especially through data augmentation. One of the notable outcomes was the model’s adeptness in identifying and extracting vital information, notably from the layer with the second-lowest density, housing 100 neurons, essential features imperative for detecting lung diseases. The DCNN model, depicted in Figure 9, showcases the process of feature extraction.

The model shown in Table 3 has four convolutional layers, each with varying filter counts (8, 16, 32, and 64). These layers use ReLU activation and batch normalization. Max-pooling layers with a pool size of 2 × 2 follow each convolutional layer. The compressed output is fed into two compact layers; the first layer consists of 128 features and a dropout, while the subsequent layer consists of 100 features. ReLU activation, batch normalization, and dropout regularization are seen in both dense layers.

#### 3.4.2. GNN Model

The GNN framework is a deep learning method for graph-based data. It uses deep learning to look at graph data by capturing each node’s local structure and features [58]. These models are trainable and generate highly informative node representations, demonstrating substantial success in tackling machine learning challenges associated with graph data. The fundamental aim of GNN architecture is to extract knowledge-rich embeddings containing pertinent information from the local neighborhood [59]. To predict lung diseases, a GNN model amalgamates a five-layer feedforward network (FFN) with a two-layer GCN [60]. The initial layer integrates batch normalization to ensure stable performance and counteract overfitting. Additionally, to bolster model generalization and avert excessive reliance on individual nodes, a dropout rate of 0.2 is introduced in the second layer. Addressing the issue of ‘vanishing gradients’, common in deep neural networks, the third layer employs a dense architecture activated by ReLU [61]. The culmination of these elements guarantees the development of a robust and efficient predictive model for lung diseases.

#### 3.4.3. Proposed GNN Architecture

In the context of a graph G=(V,E), where *V* represents the set of nodes and *E* represents the set of edges, an attributed graph encompasses two distinct forms of information [58]. The first type is the structure information, which characterizes the interconnections between nodes. The second type is the node feature information, which details the characteristics associated with each node. The input of the graph G=V,E is graph_info = (node_features, edges, edge_weights), where the nodes are represented by $x_{v}$ and edge weights $w_{ij}$ indicate the link strength between nodes *i* and *j* [62].

(8) $$r_{v}^{\left(0\right)}=\mathrm{FFN}\text{\_}\mathrm{pre}\left(x_{v}\right)$$

The initial representation of *a* node *v*, denoted as $r_{v}^{\left(0\right)}$, is based on its feature $x_{v}$. This representation is processed using an FFN called FFN_pre for preprocessing.

(9) $$r_{v}^{\left(k+1\right)}={\mathrm{GCN}}_{k}\left(r_{v}^{\left(k\right)}\;\mathrm{AGGREGATE}\left(r_{v}^{\left(k\right)},M_{v}\right)\right)+r_{v}^{\left(k\right)}$$

(10) $$r_{v}^{\left(k+1\right)}={\mathrm{GCN}}_{k}\left(r_{v}^{\left(k\right)}\right)\;\mathrm{UPDATE}\left(r_{v}^{\left(k\right)},M_{v}\right)$$

The $GCN_{k}$ represents the graph convolutional layer at a specific depth *k*. The function AGGREGATE combines the embeddings of neighboring nodes, $M_{v}$ for a given node. Addition signifies a skip connection in the network. The variable $r_{v}^{\left(k+1\right)}$ represents the revised representation of node *v* at iteration (k+1). The previous representation at iteration is denoted as $r_{v}^{\left(k\right)}$. The function UPDATE merges the previous representation with a message mv in order to generate the updated representation.

(11) $$z_{v}=\mathrm{FFN}\text{\_}\mathrm{post}\left(r_{v}^{\left(k\right)}\right)$$

The variable k is used to denote the number of graph convolutional layers in the model. The final embedding of node v is denoted as $z_{v}$, whereas the FFN utilized for post-processing is referred to as FFN_post.

(12) $$Z=\mathrm{softmax}(B\cdot \mathrm{ReLU}\left(AY^{0}\right)Y^{1})$$

The equation shows a GNN layer, where B is the normalized adjacency matrix, *X* is made of node properties, $Y^{0}$ and $Y^{1}$, and its weight matrices. Combining the traits of nodes with the structure of the graph, the equation puts them through nonlinear transformations to make predictions or embeddings. The ReLU function adds nonlinearity, while the softmax function assures that the output values are between 0 and 1, which is commonly employed for classification tasks.

**Figure 10:**
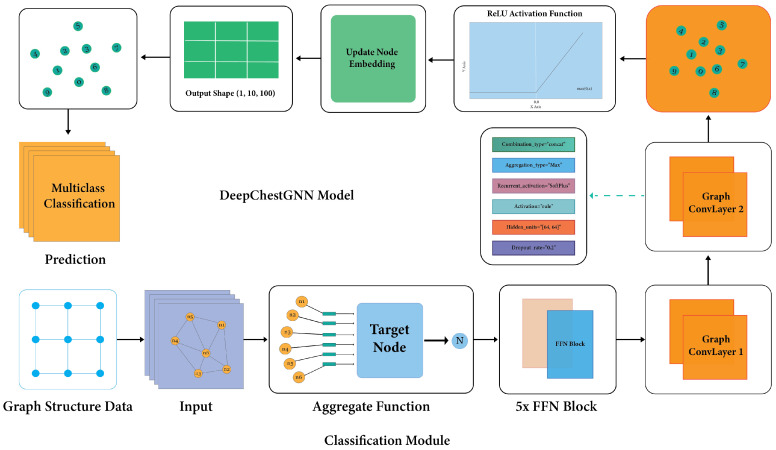
Proposed model (DeepChestGNN) architecture.

The GNN model selects neighboring nodes and combines their traits with aggregation functions. The target node is identified and passed through an FFN block, with the output forwarded to the GCN layer, which updates nodes and transmits messages to another convolution layer. Each layer of the model encompasses crucial components: activation function, activation type, recurrent activation, hidden unit, and dropout rate. Experiments showed that the best design for the model is a five-block FFN that subsequently predicts the result in the dense layer by utilizing the output from the two GCN layers, which effectively captures the complex dependencies and interactions within the graph structure. Node embedding data produce a metric from the dense layer, facilitating model outcome computation. The ReLU is employed as the activation function. The CONCAT combination type handles temporal information in sequential data, enhancing the model’s capabilities. The architectural design of the GNN model is depicted in Figure 10, guiding the model’s operation and development through experiments with varied dataset configurations. The model is configured with two hidden layers containing 64 units, enabling intricate data representation analysis and pattern discernment. The model undergoes training for 100 epochs, utilizing the Adam optimizer with a learning rate of 0.0001. Batches of size 64 and a dropout rate of 0.2 are applied during the training process.

### 3.5. Dataset Split

After obtaining the preprocessed chest X-ray images, we divided them into training and testing sets with a 70:30 ratio. The dataset comprises 70,000 images depicting ten different diseases, divided into a training set of 49,000 images used for fine-tuning and training GNN models. The test set consists of 21,000 images to evaluate the model’s performance and generalization ability to new data.

### 3.6. Experimental Setup

In our experimental setup, we conducted our research on a desktop computer with the following specifications. This study operates on an Intel(R) Core(TM) i5-8400 CPU running at 2.80 GHz (Intel, Santa Clara, CA, USA), equipped with 16.0 GB of RAM. The system operates on a 64-bit operating system with an x64-based processor architecture. To accelerate computational tasks, an NVIDIA GeForce GTX 1660 GPU (NVIDIA, Santa Clara, CA, USA) is employed. All experiments are conducted within the Jupyter Notebook environment using version 6.4.12. In some cases, we used CollabPro as a platform for running experiments when we faced difficulties with computation.

## 4. Result and Discussion

### 4.1. Evaluation Metrics

This section used a set of evaluation metrics to determine how well the models work. The confusion matrix is an important part of this evaluation because it gives important numbers like true positive (TP), true negative (TN), false positive (FP), and false negative (FN) [62]. TP means that the positive class prediction was correct, while FP means that the positive class prediction was wrong. FN stands for the wrong identification of the negative class, and TN for the correct identification of the negative class. The model’s accuracy, precision, recall, and F1-score are evaluated based on these values, with precision indicating the model’s ability to differentiate between correctly classified samples and those in the positive class. Furthermore, precision and recall are combined to derive the F1-Score, a comprehensive metric that assesses the model’s overall efficiency. In contrast, accuracy captures the total number of samples correctly categorized across all classes [63]. When the prediction is sufficient across all four categories of the confusion matrix (i.e., TP, TN, FP, and FN), the Matthews correlation coefficient (MCC) produces excellent results, making it a more reliable statistical measure [64]. We computed precision, recall, F1-score, and accuracy using Equations (13)–(17) to assess the performance for chest X-ray diseases.

(13) $$Precision=\frac{TP}{TP+FP}$$

(14) $$Recall=\frac{TP}{TP+FN}$$

(15) $$F1-Score=2*\frac{Recall*Precision}{Recall+Precision}$$

(16) $$Accuracy=\frac{TP+TN}{TP+TN+FP+FN}$$

(17) $$\mathrm{MCC}=\frac{\left(TP\times TN-FP\times FN\right)}{\sqrt{\left(TP+FP)(TP+FN)(TN+FP)(TN+FN\right)}}$$

### 4.2. Ablation Study

Ablation experiments optimized the proposed model by testing feature reconstruction algorithms, extractors, GNN layers, FFN blocks, and model hyperparameters shown in Table 4 and Table 5. These experiments fine-tuned the model’s settings for optimal performance in various scenarios and configurations [48].

In Ablation Study 1, our investigation into GNN layer depth revealed a delicate balance between model complexity and performance. A single GNN layer achieved a precision rate of 95.38%, while a dual-layered structure reached a high of 97.35%. However, a third GNN layer slightly decreased accuracy to 96.67%. The optimal balance was found with a bi-layered GNN architecture. In Ablation Study 2, FFN block configurations were examined, and subtle performance variations were showcased. The accuracy rate increased from 96.27% in a two-block configuration to 97.35% with three blocks. The five-block configuration demonstrated peak performance at 98.79%, emphasizing the effectiveness of a quintet FFN block setup in maximizing model performance.

Ablation Study 3 investigated the impact of batch size on model convergence and computational efficiency, evaluating sizes from 16 to 128 batches. The optimal batch size for further study was 64, achieving the highest accuracy of 99.06%, making it suitable for subsequent ablation studies. In Ablation Study 4, the analysis of dropout rates revealed nuanced performance gradients. A dropout rate of 0.2 emerged as the most effective for regularization, yielding 99.06% accuracy. However, an increase to 0.3 led to a decline in accuracy to 98.11%. This underscores the importance of a 0.2 dropout rate, striking a balance between regularization and model performance, making it the recommended choice for ongoing evaluations. In Ablation Study 5, adjustments to loss functions yielded varying accuracy impacts, with categorical cross-entropy achieving the highest accuracy at 99.27%, surpassing the previous 99.06% with binary cross-entropy. Mean squared error and mean absolute error decreased accuracy to 98.83% and 98.13%, respectively. Ablation Study 6, exploring optimizer variations, showed Adam topping at 99.27%, while Adamax, RMSprop, and Nadam experienced accuracy drops at 99.05%, 98.93%, and 98.82%, respectively. Ablation Study 7, focusing on learning rates, identified 0.0001 as the optimal rate with the highest accuracy of 99.74%, outperforming other rates like 0.1, 0.5, 0.001, 0.005, and 0.0005. The recommendation for further experimentation is the optimal learning rate of 0.0001. This comprehensive analysis provides valuable insights into the impact of different configurations on the proposed model’s accuracy and performance.

### 4.3. Comparison Performance Analysis of Different Feature Extractor Models

First, we compared our proposed feature extractor model, the DCNN model, with some different deep feature extractors. The comparison in Table 6 highlights its superior predictive capabilities compared to well-known models, where the highest accuracy was achieved by VGG16 with 93.32%, the second highest was DenseNet121 with 91.58%, and the lowest accuracy was achieved by ResNet50 with 89.55%. Our proposed DeepChestGNN classification model was tested against other feature extractors, revealing that our DCNN model was superior in classifying chest X-ray images, achieving a higher accuracy rate of 99.74%. This observation suggests its potential utility for reliable diagnostic purposes.

### 4.4. Comparison Performance Analysis of Different Deep Learning Models

In our comparison of our proposed model with different deep CNN models, this remarkable achievement positions it as a superior predictor compared to well-known models like Inception, which achieved the best accuracy score of 90.35%, and the second-best score was VGG16, at 89.52%. On the contrary, DenseNet201 obtained the lowest accuracy score of 83.38%, as detailed in Table 7. Our proposed DeepChestGNN emerged as the leading performer, with an exceptional accuracy rate of 99.74%. These results affirm the reliability and efficacy of DeepChestGNN in our study and underscore its potential for making accurate predictions about chest X-ray images, solidifying its status as the best model in this field.

### 4.5. Comparison Performance of Proposed Models with Other Literature

Table 8 presents a comprehensive comparison between our proposed DeepChestGNN model and several shallow GNN models from other works in the literature. The sensitivity, precision, F1-Score, and accuracy metrics were evaluated for each model. In contrast, existing models like NSCGCN [17], PNA-GCN [9], ResGNet-C [65], Efficient-B4-FPN [66], and GraphSAGE [67] demonstrate lower accuracy levels ranging from 63.83% to 98.04%. The highest accuracy was achieved by NSCGCN [17] at 98.04% and by PNA-GCN [9] at 97.79%, demonstrating the second-highest accuracy. GraphSAGE [67] achieved the lowest accuracy at 63.83% in the classification results. Notably, DeepChestGNN outperforms other models with exceptional metrics: sensitivity of 98.72%, precision of 98.71%, F1-Score of 98.71%, and an impressive accuracy of 99.74%. These results highlight the substantial improvement and effectiveness of our DeepChestGNN in comparison to established algorithms, emphasizing its potential for accurate chest X-ray image predictions in a medical context.

### 4.6. Classification Performance of DeepChestGNN Model

The performance metrics for the DeepChestGNN model’s disease classification are shown in Table 9. The model did a great job with a wide range of diseases, exhibiting high sensitivity, specificity, precision, negative predictive value (NPV), accuracy, and F1-score. It was amazing how well our proposed model worked with a wide range of diseases, with accuracy levels above 99% for some of them. It was especially good at identifying diseases like COVID-19 (97.54%), pneumonia (99.12%), pneumothorax (99.57%), and tuberculosis (99.33%), which demonstrates its sensitivity and accuracy. The model also showed high identification for lung opacity (97.88%), nodules (99.01%), and effusion (98.40%). The Matthews correlation coefficient (MCC) value for tuberculosis had the highest accuracy at 99.31%, followed by pneumothorax accuracy at 99.32%, and the lowest accuracy at 97.99%. These outstanding results indicate that the model is reliable and proficient in diagnosing various chest diseases, making it a robust tool for medical image analysis.

### 4.7. Implementation Details

**Figure 11:**
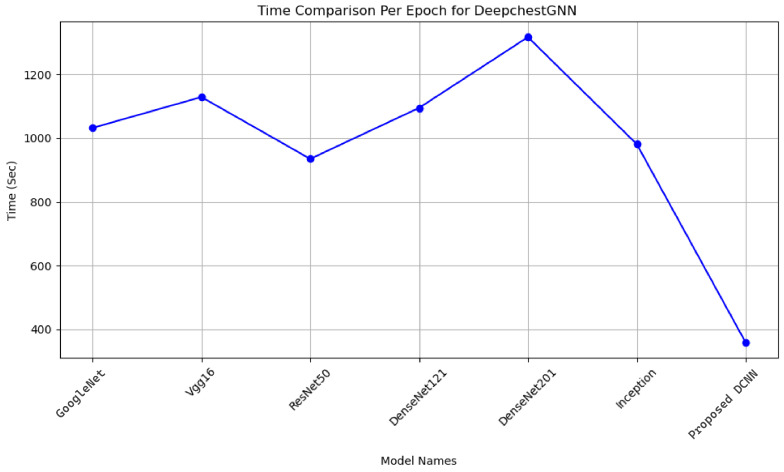
Time comparison among our proposed DeepChestGNN model with our DCNN extractor and different feature extractors.

Different feature extractors were tested in our DeepChestGNN model, revealing that our DCNN exhibited greater accuracy and required less time to execute compared to others. This observation suggests its potential utility in classifying chest X-ray images for diagnostic purposes. Each feature extractor model’s time required per epoch was meticulously measured, including GoogleNet, VGG16, ResNet50, DenseNet121, DenseNet201, Inception, and our proposed DCNN. In contrast, the execution times for integrating various feature extractors into our model per epoch were as follows. GoogleNet was executed in 1015 s, VGG16 in 1120 s, ResNet50 in 920 s, DenseNet121 in 1080 s, DenseNet201 in 1320 s, and Inception in 990 s. Our proposed DCNN feature extractor model, integrated with our proposed DeepChestGNN, demonstrated the most efficient performance, executing in just 359 s per epoch. This extraction process was conducted over 100 epochs, as illustrated in Figure 11. Such information is essential for researchers and practitioners seeking to optimize workflow extractors for medical image analysis.

### 4.8. Confusion Matrix and ROC Curve of the Proposed Model

**Figure 12:**
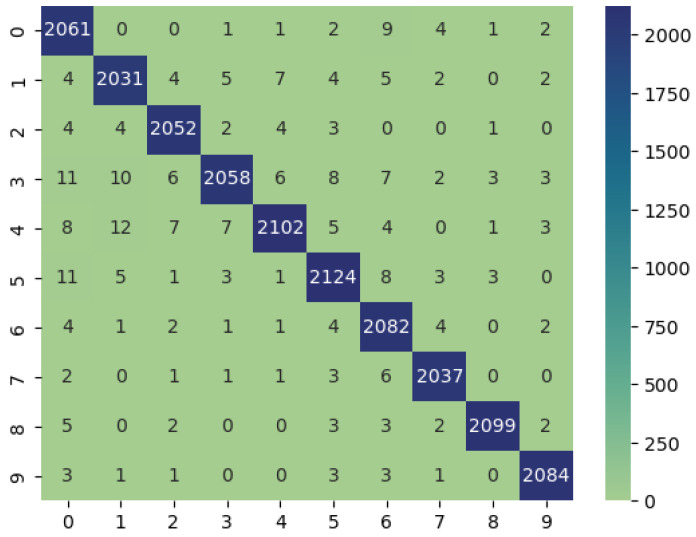
Confusion matrix of DeepChestGNN model.

**Figure 13:**
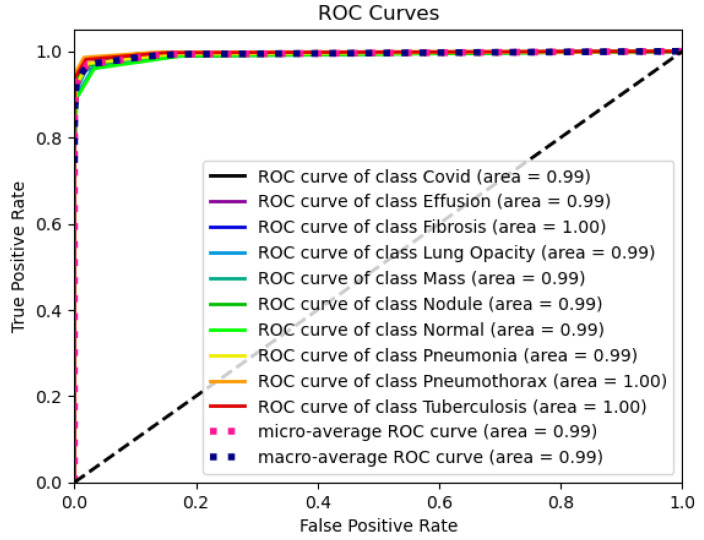
ROC curve of the DeepChestGNN model.

The confusion matrix in Figure 12 shows how the DeepChestGNN classifier consistently produces higher true positive predictions for all ten lung disease classes while maintaining an exceptionally low rate of false predictions across each category [48]. This signifies that the model exhibits an absence of bias towards any specific disease class and demonstrates its capability to predict all classes effectively [63]. Notably, our proposed DeepChestGNN model consistently achieved optimal accuracy of over 99% for all ten categories of lung diseases, as shown in Figure 13. This proves the model is reliable and consistent in predicting a wide range of lung diseases.

### 4.9. K-Fold Cross-Validation Analysis

**Figure 14:**
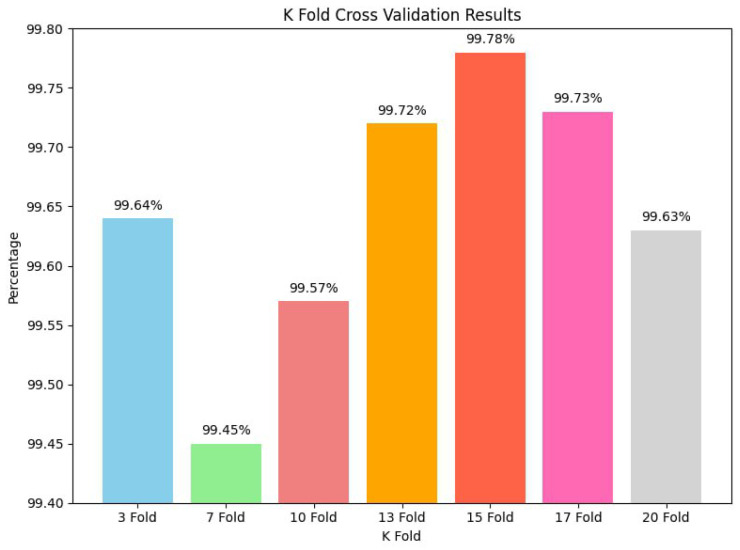
Performance analysis using K-fold cross-validation.

K-fold cross-validation is a pivotal validation technique used to evaluate the robustness and reliability of machine learning models [48]. In this study, we conducted a series of K-fold cross-validation experiments to assess the performance of our model. We tested 3-fold, 7-fold, 10-fold, 13-fold, 15-fold, 17-fold, and 20-fold cross-validation. Each K-fold cross-validation iteration divided the dataset into K subsets, with one used for validation and the rest for training. We had 99.64% testing accuracy for 3-fold, 99.45% for 7-fold, 99.57% for 10-fold, 99.72% for 13-fold, 99.78% for 15-fold, 99.73% for 17-fold, and 99.69% for 20-fold. Our best model had the highest testing accuracy of 99.78% in our experiments. These results demonstrate our model’s impressive stability and reliability across various K-fold cross-validation scenarios. Even with different K-fold settings, the model consistently maintained high accuracy, reinforcing our confidence in its performance and generalization capabilities [63]. Based on these results, the proposed model is very strong and flexible, which means it can be used in a wide range of training situations with the dataset. Figure 14 graphically displays the results, demonstrating the testing accuracy across different K-fold values, validating the model’s overall reliability and consistent performance.

## 5. Visualization Results

### 5.1. Experiment with Original Images

**Figure 15:**
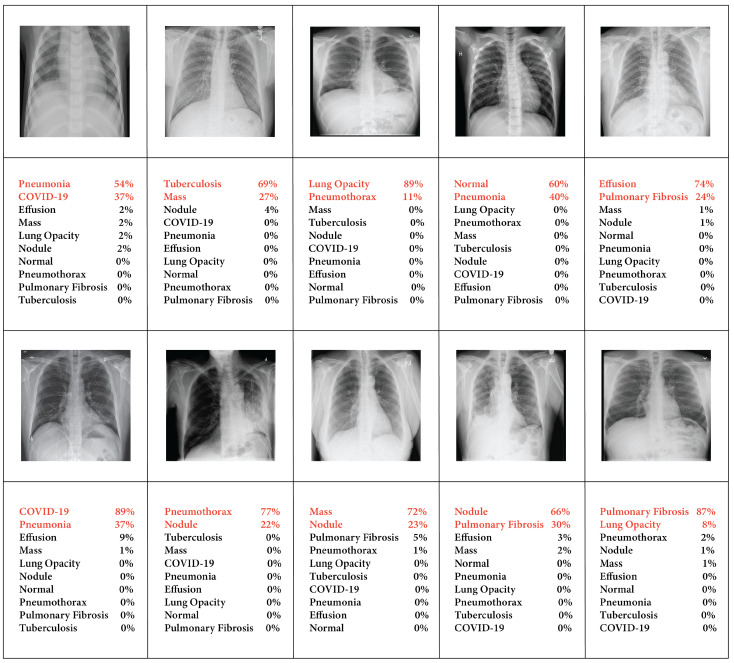
Our dataset’s raw images without preprocessing show predicted categories and probability scores, highlighted in red, indicating a higher likelihood of the associated disease.

Figure 15 presents a comprehensive multi-class chest X-ray image classification visualization. The diagram illustrates prediction scores for each test image, with the highest values at the top, providing a brief overview of the model’s performance on original images. For instance, the model’s performance across various classes showed that the highest accuracy was achieved for lung opacity at 89%, COVID-19 at 89%, and fibrosis at 87%, demonstrating the second-highest accuracy. In contrast, pneumonia had the lowest accuracy of 54% in prediction. This accuracy was achieved when the unprocessed image was passed into our automated system without applying any image pre-processing techniques, indicating that image processing was required for better accuracy.

### 5.2. Experiment with Adding Noise

**Figure 16:**
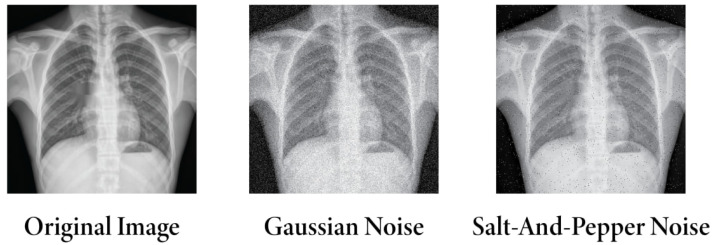
Differences among original image, Gaussian noise, and salt-and-pepper noise images.

As the dataset was compiled from multiple sources, image pre-processing in large data hubs was necessary to obtain the highest precision in classification. Initially, denoising, resizing, de-annotation, enhancement, and applying filters were employed to remove noise and overlay text. Subsequently, these preprocessed images were subjected to our proposed method. However, Gaussian noise [68] and salt-and-pepper noise [69] were added to the original dataset in more tests to see how well the model could evaluate robustness and generalization. The noisy images are illustrated in Figure 16.

After adding noise and subsequently applying our DCNN feature extractor and DeepChestGNN, a slight decrease in accuracy was observed compared to the original image of 99.74%. The accuracy decreased to 97.57% for images affected by Gaussian noise and to 98.04% for images affected by salt-and-pepper noise. All the results are shown in Table 10. Despite the reduction in accuracy, the effect of noise on overall performance was relatively small. However, these minor abnormalities significantly affect clinical outcomes in diagnosis. In this regard, employing specialized image-processing methods is crucial. This study achieved the highest accuracy after applying preliminary image pre-processing steps, which strongly contributed to removing noise artifacts and other distortions, resulting in high accuracy.

## 6. Comparison with Several Existing Studies

The main objective of our work in this section was to compare our work with existing the literature. Table 11 presents a comparative analysis between prior studies and our proposed method, evaluating factors such as the number of images, the number of features, the effectiveness of image preprocessing and augmentation methods, and overall accuracy. In binary classifications [9,14,16,17,20,21], Liang et al. [19] achieved an impressive highest accuracy of 98.5% for COVID-19 and normal, while Jin et al. [16] recorded a comparatively lower accuracy of 80.0% for COVID-19 and non-COVID-19. For multi-class classification [5,8,10,11,12,13,15,20], Nahiduzzaman et al. [15] addressed 17 classes but did not achieve remarkable accuracy, reaching only 90.92%. Kufel et al. [12] achieved a lower accuracy of 84.28% for classifying fifteen classes of chest X-ray images. Moreover, [9,12,13,15,16,17,18,19,20] did not address the absence of noise and overlay text removal. There is also a lack of information on optimal features and low accuracy in multi-class classification, as shown in Table 11. To address those limitations, an automated system was employed. The study leveraged multiple resources, comprising 71,096 images, and utilized image preprocessing techniques such as resizing, denoising, CLAHE, de-annotation, and filtering. Elastic deformation was applied for augmentation in the dataset, which started with 70,000 images. Additionally, the model demonstrated efficient processing time, with one epoch taking only 359 s, attributed to having only 100 prominent deep features compared to other models. The multi-class classification model employed was the proposed DeepChestGNN, addressing ten classes, achieving an impressive accuracy of 99.74%. Furthermore, we introduced a strategy based on these optimal features and successfully validated it using real images for diagnosis.

## 7. Limitations and Future Research

Our automated system has already showcased superior accuracy and computational efficiency across diverse datasets, effectively managing variations in classes, noises, and overlaying complexities in chest X-rays. This success points towards a promising future for medical imaging technology. Our upcoming studies will prioritize expanding our dataset and subjecting our model to a broader range of conditions, focusing on real-time chest X-ray images. We plan to explore the potential of graph convolutional networks (GCNs) and generative adversarial networks (GANs) to enhance dataset robustness. Additionally, we aim to develop a computer-based decision-making tool to assist medical professionals in treating patients with lung diseases. An essential enhancement in our pipeline involves prioritizing image segmentation, potentially improving feature extraction. Ultimately, we aim to create a real-time lung disease classification application, seamlessly integrating academic research with clinical applications and providing medical professionals with a more efficient diagnostic tool.

## 8. Conclusions

In this study, we employed an automated system for classifying lung diseases using a large data hub consisting of 17 chest X-ray image datasets. The ten disease categories in the large data hub are normal, effusion, pulmonary fibrosis, lung opacity, mass, nodule, COVID-19, pneumonia, pneumothorax, and tuberculosis. For collecting data from various resources, data with noise or overlay texts, etc., we employed image processing techniques such as resizing all the images into the same 420 × 420 pixel size, denoising for reducing noise, and CLAHE while maintaining the tile grid size at (8 × 8) and the clipping limit at 3.0. To enhance the quality of images, we utilized the ‘Green Fire Blue’ filter to emphasize specific image attributes. Elastic deformation augmentation enhances image quality and addresses class data imbalances, resulting in a well-trained model with a dataset volume of 70,000 images. The DCNN feature extractor demonstrated its ability to identify 100 essential deep features accurately. Our proposed DeepChestGNN model exhibits a well-structured design with batch normalization, MaxPooling, and Dropout for regularization, striking a balance between expressiveness and overfitting avoidance. A meticulous exploration through ablation studies led to the optimal model configuration, incorporating a bi-layered GNN architecture, a five-block FFN structure, a batch size of 64, a dropout rate of 0.2, categorical cross-entropy as the loss function, and the Adam optimizer with a learning rate of 0.0001. We also achieved an impressive accuracy of 99.74%. This research underscores the transformative potential of seamlessly integrating cutting-edge image-processing techniques with advanced deep learning models in medical diagnostics. The remarkable promise exhibited by DeepChestGNN signifies a groundbreaking leap toward ensuring timely and precise diagnoses of diverse lung diseases through the analysis of chest X-ray images.

## Figures and Tables

**Table 1:** The number of images corresponding to the ten classes of the seventeen datasets.

No.	Name of Class	Number of Images	References
1	Normal	13,953	[22,23,26,27,29,36]
2	Tuberculosis	5242	[22,30,32,36]
3	Lung Opacity	7212	[24,29]
4	COVID-19	11,566	[24,26,27,29,31]
5	Pneumonia	11,683	[23,24,26,32]
6	Pneumothorax	6111	[25,33,37]
7	Nodules	4109	[25,28,38]
8	Fibrosis	2798	[25,28,32]
9	Effusion	5527	[25,28,35,35]
10	Mass	2895	[25,28]
Total number of images		71,096	[22,23,24,25,26,27,28,29,30,31,32,33,34,35,36,37,38]

**Table 2:** All of the characteristics of the final dataset (after image pre-processing).

Name of Elements	Properties
Number of Images	70,000
Number of Classes	10
Enhancement and Color Grading	CLAHE, Green Fire Blue
Augmentation Techniques	Elastic Deformation
Normal	7000
Tuberculosis	7000
Lung Opacity	7000
COVID-19	7000
Pneumonia	7000
Pneumothorax	7000
Nodules	7000
Fibrosis	7000
Effusion	7000
Mass	7000

**Table 3:** Feature extraction model layout.

Layers	Parameters	Activation Function, Batch Normalization Function, Dropout	Output Shape
Conv2D_1	Kernel Size: 3 × 3	ReLU, Yes, No	(222, 222, 8)
MaxPooling2D_1	Kernel Size: 2 × 2	None	(111, 111, 8)
Conv2D_2	Kernel Size: 3 × 3	ReLU, Yes, No	(109, 109, 16)
MaxPooling2D_2	Kernel Size: 2 × 2	None	(54, 54, 16)
Conv2D_3	Kernel Size: 3 × 3	ReLU, Yes, No	(52, 52, 32)
MaxPooling2D_3	Kernel Size: 2 × 2	None	(26, 26, 32)
Conv2D_4	Kernel Size: 3 × 3	ReLU, Yes, No	(26, 26, 64)
MaxPooling2D_4	Kernel Size: 2 × 2	None	(12, 12, 64)
Flatten Layer	None	None	(10,816)
Dense_1	None	ReLU, Yes, Yes	(128)
Dense_2	None	ReLU, Yes, Yes	(100)

**Table 4:** Ablation study regarding GNN layer and FFN block.

**Ablation Study 1: Altering the GNN Layer**			
**Configuration No.**	**GNN Layer**	**Test Accuracy (%)**	**Finding**
1	1	95.38	Accuracy dropped
2	2	97.35	Highest Accuracy
3	3	96.67	Accuracy dropped
**Ablation Study 2: Altering the FFN Block**			
**Configuration No.**	**FFN Block**	**Test Accuracy (%)**	**Finding**
1	2	96.27	Accuracy dropped
2	3	97.35	Previous Accuracy
3	4	97.13	Accuracy dropped
4	5	98.79	Highest Accuracy
5	6	97.48	Accuracy Improved

**Table 5:** Ablation study regarding model hyperparameters and loss function.

**Ablation Study 3: Altering the Batch Size**			
**Configuration No.**	**Batch Size**	**Test Accuracy (%)**	**Finding**
1	16	98.13	Accuracy dropped
2	32	98.79	Previous Accuracy
3	64	99.06	Highest Accuracy
4	128	98.42	Accuracy dropped
**Ablation Study 4: Altering the Dropout Rate**			
**Configuration No.**	**Dropout rate**	**Test Accuracy (%)**	**Finding**
1	0.1	98.29	Accuracy dropped
2	0.2	99.06	Previous Accuracy
3	0.3	98.11	Accuracy dropped
**Ablation Study 5: Altering the Loss Functions**			
**Configuration No.**	**Loss Functions**	**Test Accuracy (%)**	**Finding**
1	Binary cross-entropy	99.06	Previous Accuracy
2	Categorical cross entropy	99.27	Highest Accuracy
3	Mean squared error	98.83	Accuracy dropped
4	Mean absolute error	98.13	Accuracy dropped
**Ablation Study 6: Altering the Optimizer**			
**Configuration No.**	**Optimizer**	**Test Accuracy (%)**	**Finding**
1	Adam	99.27	Previous Accuracy
2	Adamax	99.05	Accuracy dropped
3	RMSprop	98.93	Accuracy dropped
4	Nadam	98.82	Accuracy dropped
**Ablation Study 7: Altering the Learning Rate**			
**Configuration No.**	**Learning Rate**	**Test Accuracy (%)**	**Finding**
1	0.1	98.53	Accuracy dropped
2	0.5	98.27	Accuracy dropped
3	0.001	98.68	Accuracy dropped
4	0.005	98.79	Accuracy dropped
5	0.0001	99.74	Highest Accuracy
6	0.0005	99.27	Previous Accuracy

**Table 6:** Performance of DeepChestGNN (ours) based on our DCNN extractor and different feature extractors.

Model	Sensitivity	Precision	F1-Score	Accuracy
Inception	93.85%	93.94%	93.86%	93.85%
VGG16	93.32%	93.54%	93.32%	93.32%
DenseNet121	91.53%	91.68%	91.55%	91.58%
DenseNet201	90.32%	90.45%	90.32%	90.35%
GoogleNet	90.56%	90.62%	90.55%	90.55%
ResNet50	89.56%	90.13%	89.55%	89.55%
DCNN (ours)	98.72%	98.71%	98.71%	99.74%

**Table 7:** Comparison of DeepChestGNN (ours) with different deep CNN model.

Model	Sensitivity	Precision	F1-Score	Accuracy
Inception	90.34%	90.45%	90.38%	90.35%
VGG16	89.52%	89.78%	89.53%	89.52%
GoogleNet	87.44%	87.54%	87.44%	87.45%
DenseNet121	84.45%	84.51%	84.45%	84.45%
ResNet50	83.95%	84.10%	83.95%	83.95%
DenseNet201	83.38%	83.45%	83.37%	83.38%
DeepChestGNN (ours)	98.72%	98.71%	98.71%	99.74%

**Table 8:** Comparison between shallow GNN models and DeepChestGNN (ours) model.

Model	Sensitivity	Precision	F1-Score	Accuracy
NSCGCN [17]	87.50%	87.37%	87.50%	98.04%
PNA-GCN [9]	95.43%	98.51%	98.63%	97.79%
ResGNet-C [65]	95.91%	96.65%	96.21%	96.62%
Efficient-B4-FPN [66]	91.01%	89.13%	95.96%	95.36%
GraphSAGE [67]	63.83%	76.59%	58.90%	63.83%
DeepChestGNN (ours)	98.72%	98.71%	98.71%	99.74%

**Table 9:** Performance evaluation of the DeepChestGNN (ours) model corresponding to the classes.

Disease	Sensitivity	Specificity	Precision	NPV	Accuracy	F1-Score	MCC
COVID-19	99.04%	99.73%	97.54%	99.89%	99.66%	98.28%	98.10%
Effusion	98.40%	99.83%	98.40%	99.83%	99.69%	98.40%	98.23%
Fibrosis	99.13%	99.87%	98.84%	99.90%	99.80%	98.99%	98.88%
Mass	97.35%	99.89%	99.04%	99.70%	99.64%	98.19%	97.99%
Nodule	97.81%	99.89%	99.01%	99.75%	99.68%	98.41%	98.23%
Normal	98.38%	99.81%	98.38%	99.81%	99.67%	98.38%	98.19%
Lung Opacity	99.10%	99.76%	97.88%	99.90%	99.70%	98.49%	98.32%
Pneumonia	99.32%	99.91%	99.12%	99.93%	99.85%	99.22%	99.10%
Pneumothorax	99.20%	99.95%	99.57%	99.91%	99.88%	99.38%	98.32%
Tuberculosis	99.43%	99.93%	99.33%	99.94%	99.88%	99.38%	98.31%

**Table 10:** Performance comparison of different noise types with the original image classes.

Noise Type	Sensitivity	Specificity	Precision	NPV	Accuracy	F1-Score	MCC
Gaussian Noise	96.06%	96.59%	96.92%	97.13%	97.57%	97.23%	96.98%
Salt-and-Pepper Noise	96.83%	97.12%	97.52%	97.89%	98.04%	96.02%	95.81%
Original Image (prepossessed image)	98.72%	99.86%	98.71%	99.86%	99.74%	98.71%	98.57%

**Table 11:** Accuracy comparison between our proposed work and existing literature.

Paper	Dataset	Model	Classification Types	Accuracy	Limitations
Sanida et al. [10]	COVID-19 Radiography Database (21,165 images)	Preprocessing: Applied Augmentation: Yes Feature Extraction: N/A Model: Modified VGG19	Multi-class classification—(fibrosis, opacity, tuberculosis, normal, viral pneumonia, and COVID-19 pneumonia)	Accuracy: 98.88% Feature num: More than 100	The limited number of images. Lack of noise and overlay text removal from images. Absence of information on optimal features. Lack of comparison with more recent state-of-the-art methods
Abubakar et al. [11]	CT image datasets (328 common pneumonia, 1972 COVID-19, and 1608 healthy images)	Image Preprocessing: Applied Augmentation: Yes Feature Extraction: HOG and CNN Model:KNN, SVM	Multi-class classification (COVID-19-positive, healthy, and common pneumonia)	Accuracy: VGG-16 + HOG feature achieved 99.4% overall accuracy with SVM Feature num: More than 100	The limited number of images Lack of noise and overlay text removal from images. Absence of information on optimal features. Lack of comparison with more recent state of-the-art methods.
Kufel et al. [12]	NIH ChestX-ray14 (112,120 images)	Image Preprocessing: N/A Augmentation: Yes Feature Extraction: EfficientNet Model: Transfer learning techniques	Multi-class (15 classes—No Finding, Atelectasis, Cardiomegaly, Effusion, Infiltration, Mass, Nodule, Pneumonia, Pneumothorax, Consolidation, Edema, Emphysema, Fibrosis, Pleural thickening, Hernia)	Accuracy: 84.28% Feature num: More than 100	Lack of noise and overlay text removal from images. Absence of information on optimal features. Low accuracy in multi-class classification.
Li et al. [13]	1. ChestX-Ray 14 (112,120 images) 2. CheXpert (224,316 image)	Image Preprocessing: N/A Augmentation: Yes Feature Extraction: Res2Net50 Model: MLRFNet	Multi-class (7 classes—Atelectasis, Effusion, Infiltration, Mass, Nodule, Pneumonia, Pneumothorax)	Accuracy: 1. 85.30% 2. 90.40% Feature num: More than 100	Lack of noise and overlay text removal from images. Absence of information on optimal features. Low accuracy in multi-class classification.
Farhan et al. [14]	1. COVID-19 Radiography Database (C19RD) (2905 images) 2. Chest X-ray Images for Pneumonia (CXIP) (5856 images)	Image Preprocessing: Applied Augmentation: No Feature Extraction: Res2Net50 Model: HDLA-DNN classifier	Binary classification—disease (such as non-COVID-19 pneumonia, COVID-19 pneumonia) and healthy	Accuracy: 1. 98.35% 2. 98.99% Feature num: More than 100	Lack of noise and overlay text removal from images. Absence of information on optimal features. Low accuracy in multi-class classification.
Nahiduzzaman et al. [15]	ChestX-Ray14 dataset (29,871 images)	Image Preprocessing: Applied Augmentation: No Feature Extraction: ELM Model: CNN-ELM	Multi-class (17 classes—Atelectasis, Cardiomegaly, Effusion, Infiltration, Mass, Nodule, Pneumothorax, Consolidation, Edema, Emphysema, Bacterial pneumonia, Viral pneumonia, COVID-19, Pleural thickening, Fibrosis, Hernia, and Tuberculosis)	Accuracy: 90.92% for 17 lung diseases 99.37% for COVID-19 99.98% for TB Feature num: More than 100	Lack of proper image preprocessing. Lack of a proper augmentation technique. Absence of information on optimal features. Low accuracy in multi-class classification. Lack of comparison with more recent state-of-the-art methods.
Jin et al. [16]	ChestX-ray14 (112,120 images)	Image Preprocessing: N/A Augmentation: No Feature Extraction: DenseNet121 Model: CM-DML-GZSL	Binary classification (COVID-19 and Non-COVID-19)	Accuracy: 80.0% Feature num: More than 100	Lack of noise and overlay text removal from images. Lack of a proper augmentation technique. Absence of information on optimal features. Low accuracy in multi-class classification and
Tang et al. [17]	1. CXR dataset (6939 images) 2. CT dataset (85,725 images)	Image Preprocessing: N/A Augmentation: No Feature Extraction: DenseNet201 Model: NSCGCN	Binary classification (Infection and Normal)	Accuracy: 1. 97.09% 2. 99.22% Feature num: More than 100	Lack of noise and overlay text removal from images. Lack of a proper augmentation technique. Absence of information on optimal features. Limited class classifications.
Shamrat et al. [5]	Multiple sources (Total of 85,105 images)	Image Preprocessing: Applied Augmentation: N/A Feature Extraction: N/A Model: LungNet22	Multi-class classification (10 classes-Control, COVID-19, Effusion, Lung Opacity, Mass, Nodule, Pulmonary Fibrosis, Pneumonia, Pneumothorax, Tuberculosis) After augmentation (80,000 images)	Accuracy: 98.89% Feature num: N/A	Absence of optimal features Extraction. Lack of comparison with more recent state-of-the-art methods.
Guail et al. [9]	Chest X-ray dataset from Kaggle (5856 images)	Image Preprocessing: Applied Augmentation: Yes Feature Extraction: CNN Model: PNA-GCN	Binary classification (Pneumonia and Normal)	Accuracy: 97.79% Feature num: More than 100	The limited number of images. Lack of a proper augmentation technique Absence of information on optimal features. Lack of multi-class classification.
Ragab et al. [18]	Chest X-ray dataset from Kaggle (6310 images)	Image Preprocessing: Applied Augmentation: No Feature Extraction: CNN Model: CapsNet	Multi-class classification (Pneumonia, Normal, and COVID-19)	Accuracy: 1. 86.6% for normal, 2. 94% for Pneumonia, 3. 89% for COVID-19 Feature num: More than 100	The limited number of images. Lack of a proper augmentation technique Absence of information on optimal features. Lack of multi-class classification.
Liang et al. [19]	COVID-19 (399 images) Normal (400 images)	Image Preprocessing: N/A Augmentation: No Feature Extraction: 3D-CNN Model: GCN	Binary classification (COVID-19 and Normal)	Accuracy: 98.5% Feature num: More than 100	The limited number of images. Lack of a proper augmentation technique Absence of information on optimal features. Lack of multi-class classification.
Javaheri et al. [20]	Not publicly available (16,750 slices of CT scan images from 335 patients)	Image Preprocessing: N/A Augmentation: N/A Feature Extraction: N/A Model: CovidCTNet	Binary classification (COVID-19, non-COVID-19) Multi-class classification (COVID-19, CAP, control lungs)	Accuracy: 1. 93.33% (Binary classification) 2. 86.66% (multi-class classification)	Lack of image preprocessing. Low accuracy in multiclass Classification. lack of augmentation technique.
Alshazly et al. [21]	SARS-CoV-2 CT Scan dataset (2482 images) and COVID-19-CT dataset (746 images)	Image Preprocessing: N/A Augmentation: N/A Feature Extraction: N/A Model:Different Deep learning models such as ResNet101 and DenseNet201.	Binary classification (COVID and non-COVID)	Accuracy: 1. 99.4% (ResNet101) 2. 92.9% (DenseNet201) Feature num: N/A	The limited number of images Lack of image preprocessing. Lack of comparison with more recent state-of-the-art methods.
(Our proposed work)	Multiple resources (71,096 images)	Image Preprocessing: Resizing, Denoising, CLAHE, De-annotation, Filtering Augmentation: Elastic deformation Feature Extraction: DCNN (proposed) Model: DeepChestGNN (proposed)	Multi-class classification (10 classes-Normal, Effusion, Pulmonary Fibrosis, Lung Opacity, Mass, Nodule, COVID-19, Pneumonia, Pneumothorax, Tuberculosis) After augmentation (70,000 images)	Accuracy: 99.74% Feature num: 100	The experimentation with real images is not present. Lack of pixel-level image preprocessing and segmentation using markers.

## Data Availability

Data are contained within the article.
